# Comment on Leivaditis et al. Artificial Intelligence in Cardiac Surgery: Transforming Outcomes and Shaping the Future. *Clin. Pract.* 2025, *15*, 17

**DOI:** 10.3390/clinpract15110195

**Published:** 2025-10-27

**Authors:** Hassam Ul Haq, Muhammad Abdul Haseeb Khan

**Affiliations:** Ayub Medical College, Abbottabad 22020, Pakistan; drhaseebkhan11@gmail.com

We would like to thank Leivaditis et al. and *Clinics and Practice* for the timely and comprehensive review, “Artificial Intelligence in Cardiac Surgery: Transforming Outcomes and Shaping the Future” [1]. This article provides a panoramic view of the advances and challenges associated with AI integration in cardiac surgical practice, ranging from data-driven precision medicine to the ethical, clinical, and regulatory frontiers.

Several points within the paper warrant further consideration:

**1.**  **Data Quality and Model Generalizability**

The strength of AI models is fundamentally tied to the quality and diversity of their training datasets. Many cardiac AI models are “trained” on regional or institution-specific data, yielding high internal validity but limiting their applicability to diverse patient populations and global contexts. Ensuring broad generalizability will require multi-center, prospective studies and cross-border data harmonization, consistent with emerging DECIDE-AI guidelines [2].

**2.**  **Clinical Implementation and Workflow Integration**

While the review highlights transformative applications in risk stratification, surgical planning, and intraoperative decision-making, practical integration remains an ongoing struggle. AI-driven decision support tools are often built in siloed environments, facing resistance from clinical teams due to usability issues and workflow disruptions. Robust implementation must be paired with early clinical engagement, user-centered design, and iterative feedback mechanisms.

**3.**  **Ethical, Legal, and Accountability Challenges**

Human oversight and accountability in AI-driven cardiac surgery cannot be overstated. Leivaditis et al. skillfully discuss the ethical indeterminacy—specifically, “who is responsible” when an AI-augmented system contributes to adverse outcomes. Transparent, explainable models and robust legal frameworks are needed, particularly as AI recommendations become more autonomous [3,4].

**4.**  **Addressing Algorithmic Bias and Equity**

AI in cardiac surgery, as in other specialties, risks exacerbating disparities if models reflect underlying biases in the available data. To mitigate this challenge, the development and deployment of AI should include demographic fairness audits, ongoing validation in real-world, heterogeneous cohorts, and proactive engagement with diverse patient communities [4]. The reference to algorithmic bias and its impact on vulnerable populations is particularly important and merits further research.

**5.**  **Future Directions: Collaboration and Interdisciplinary Innovation**

The field must now look beyond incremental improvements to foster cross-disciplinary partnerships, particularly with bioinformatics, legal scholars, and patient advocates. Novel AI tools, from machine learning-based risk models to robotics and virtual reality platforms, require close collaboration to enhance transparency, reliability, and clinical utility [5]. The eventual goal should be a comprehensive, human-centered ecosystem where AI augments clinician judgment rather than replaces it.

In conclusion, this review is a welcome and necessary contribution to ongoing debates in AI-driven cardiac surgery. The careful documentation of benefits, risks, and unresolved questions aligns well with *Clinics and Practice*’s mission of advancing translational science and patient care.
